# Structure-Based Peptide Design to Modulate Amyloid Beta Aggregation and Reduce Cytotoxicity

**DOI:** 10.1371/journal.pone.0129087

**Published:** 2015-06-12

**Authors:** Jitendra Kumar, Risa Namsechi, Valerie L. Sim

**Affiliations:** 1 Centre for Prions and Protein Folding Diseases, University of Alberta, Edmonton, Alberta, Canada; 2 Department of Medicine (Neurology), University of Alberta, Edmonton, Alberta, Canada; 3 Neurosciences and Mental Health Institute, University of Alberta, Edmonton, Alberta, Canada; University of Akron, UNITED STATES

## Abstract

The deposition of Aβ peptide in the brain is the key event in Alzheimer disease progression. Therefore, the prevention of Aβ self assembly into disease-associated oligomers is a logical strategy for treatment. π stacking is known to provide structural stability to many amyloids; two phenylalanine residues within the Aβ 14–23 self recognition element are in such an arrangement in many solved structures. Therefore, we targeted this structural stacking by substituting these two phenylalanine residues with their D-enantiomers. The resulting peptides were able to modulate Aβ aggregation *in vitro* and reduce Aβ cytotoxicity in primary neuronal cultures. Using kinetic analysis of fibril formation, electron microscopy and dynamic light scattering characterization of oligomer size distributions, we demonstrate that, in addition to altering fibril structural characteristics, these peptides can induce the formation of larger amorphous aggregates which are protective against toxic oligomers, possibly because they are able to sequester the toxic oligomers during co-incubation. Alternatively, they may alter the surface structure of the oligomers such that they can no longer interact with cells to induce toxic pathways.

## Introduction

Alzheimer’s disease (AD) is expected to affect 14 million North Americans by the middle of this century, yet studies have failed to find effective disease-modifying treatments. Because the accumulation of beta amyloid (Aβ) is thought to trigger AD pathogenesis[1–6], Aβ has been a logical target for therapeutic interventions[7–8]. To directly interfere with the Aβ cascade, the ideal therapeutic compound would have specificity for pathological Aβ aggregates, reduce aggregation and / or toxicity of the aggregates, be non-toxic itself, and be able to cross the blood-brain barrier.

One way to specifically prevent the accumulation of pathological Aβ, is to target the self recognition elements (SREs) that promote Aβ aggregation into toxic oligomers or fibrils. Oligomers are more toxic than fibrils, but both are associated with disease[9]. The Aβ peptide 14–23 is considered the minimal segment sufficient for fibril formation[10], with residues 17–21 involved in beta sheet formation[11]. Within oligomers of Aβ, solid state NMR has demonstrated that this 17–21 region associates intramolecularly with residues 31–36[12]. The 17–21 SRE may also be important for oligomerization of monomers, as residues 16–25 and 16–23 (for Aβ 1–40 and Aβ 1–42 respectively) have been identified as intermolecular contacts when monomers are exposed to preformed oligomers[13]. These contacts are also present in mature fibrils.[14–19]

Within the 17–21 SRE, two phenylalanines at residues 19 and 20 are positioned favourably for π-π stacking in the mature fibril. This phenylalanine stacking is fundamental to a number of amyloid structures, including fibrils of the synthetic peptide KFFEAAAKKFFE (which includes Aβ residues 19–22 (FFEA)) and of Aβ 11–25[20–21]. These aromatic residues are also known to be influential in full length Aβ aggregation[22–25] and substituting them with alanine[26–28] or proline[29] reduces or prevents fibril formation. Non-bonded interactions between the aromatic rings can provide an energetic contribution through π -stacking, as well as a specific directionality and orientation of fibril twist based on the pattern of stacking[30].

Peptide inhibitors of Aβ aggregation have been generated by modifying the amino acids within or around SREs. Of particular interest are those that include D-amino acid stereoisomers, as these peptides are more protease resistant[31], therefore more suitable for therapeutics. Aβ fragment peptides containing D-enantiomer or N-methylated amino acids have been shown to reduce Aβ aggregation [32–33]. The synthetic D-peptide klvffa (Aβ 16–21) was an effective inhibitor of Aβ aggregation, although it self-aggregated, which may not be ideal for a therapeutic agent[34]. A dipeptide construct containing D-tryptophan plus alpha-aminoisobutyric acid, which targets the aromatic residues within the SRE, improved cognitive performance in AD mouse models[35].

For this study, we created a peptide construct that targets the 17–21 SRE of Aβ and is specifically incorporated into the growing fibril to alter its structural and pathological properties. Starting with Aβ 14–23, we introduced D-enantiomer(s) in place of L-phenylalanines at positions 19 and / or 20 to sterically interfere with -stacking in the aggregate state. Our results indicate that the generated peptides do not form ThT positive aggregates, but can be incorporated into Aβ 1–42 fibrils and influence the toxicity of Aβ 1–42 oligomers, possibly by sequestering the more toxic oligomers into large amorphous aggregates.

## Results

**Fig 1:**
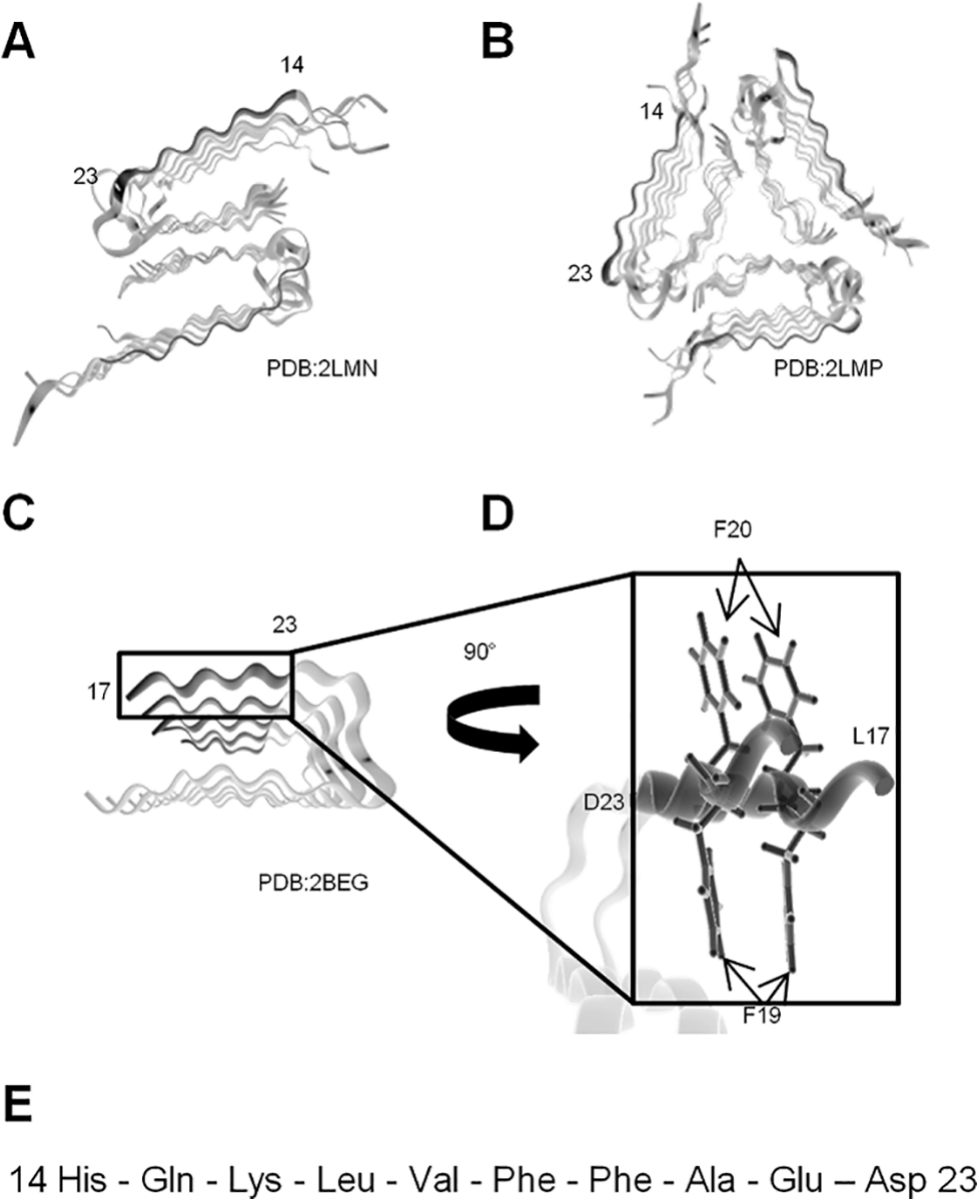
Structure of Aβ peptides 1-40/42 showing the position of residues 14–23 in the fibril formed. (A) Aβ 1–40, two fold symmetry; (B) Aβ 1–40, three fold symmetry; and (C) Aβ 1–42, two fold symmetry. (D) The position of the Phe19 and Phe20 within the PDB:2BEG structure. (E) Amino acid sequence of Aβ 14–23, the substrate for peptide substitution in this study.

Given the proposed π -stacking that occurs in the core region of the Aβ structure (Fig 1A–1C), we theorized that altering the orientation of the planar aromatic residues would interfere with fibril formation and / or the formation of a toxic species. Using the amyloidogenic region Aβ 14–23, we synthesized peptides containing D-enantiomers of the phenylalanine residues at positions 19, 20 or both 19 and 20 (Fig 1D). We refer to these substituted peptides as D19, D20 and D19/20, respectively. For a control, we used Aβ 14–23 containing only L amino acids.

### Control peptide aggregation and toxicity

The L-peptide Aβ 14–23 has been shown to readily form fibrils in 10 mM phosphate buffer, pH 7.4, when left at room temperature[11]. We confirmed the aggregation propensity of this peptide under our conditions of 10 mM phosphate buffer, pH 7.4, with 50 mM sodium chloride and incubation at 37°C (S1 Fig). The peptide was aggregate prone and had initial ThT fluorescence readings of more than 600 a.u., with no subsequent increase observed (Panel A in S1 Fig). Using electron microscopy (EM), we confirmed that fibrils were already present within minutes of starting the reaction (Panel B in S1 Fig). We interpret this to mean that aggregation is extremely efficient and rapid for this peptide under our conditions. When Aβ 1–42 was co-incubated with the L-peptide, a sigmoidal kinetic was observed, but the slope was steeper and the reaction reached completion sooner than for Aβ 1–42 alone (8.49 ± 0.18 vs 16.35 ± 1.43 hours) (Panels C and E in S1 Fig), suggesting the L-peptide enhances the aggregation efficiency of Aβ 1–42. It is well recognized that fibrils of Aβ 1–42 are much less toxic than their oligomeric form[9, 12]. The toxicity of the L-peptide fibrils was comparable to that of the Aβ 1–42 fibrils (82% vs 90% cell viability), but fibrils produced from co-incubated reactions were significantly more toxic than either alone (76% viability) (Panel G in S1 Fig).

### Aggregation kinetics

Turning to our D19, D20 and D19/20 peptides, we determined whether they would also form ThT positive aggregates under our conditions. None of the peptides formed fibrils, as measured by ThT fluorescence (S2 Fig), including during control reactions run for up to 72 hours (data not shown).

We next performed co-incubation reactions using increasing molar ratios of peptide:Aβ 1–42, where Aβ 1–42 was kept at 5 μM. With increasing ratios, a dose-dependent increase in lag phase was observed (S3 Fig). The single substitution peptides (D19 and D20) both increased lag phase at ratios as low as 0.5:1 (peptide:Aβ 1–42), whereas D19/20 only began to cause an increase in lag phase at ratios of 1:1. At 2:1 molar ratios, all peptides increased lag phase by more than 2 hours. At higher ratios, D19/20 caused a greater increase in lag phase than the single substitutions. Complete inhibition of aggregation was only achieved at a high molar ratio of 32:1 in D20 (S4 Fig). 2:1 ratios were used for all subsequent reactions, in combination with 10 μM of Aβ 1–42.

**Fig 2:**
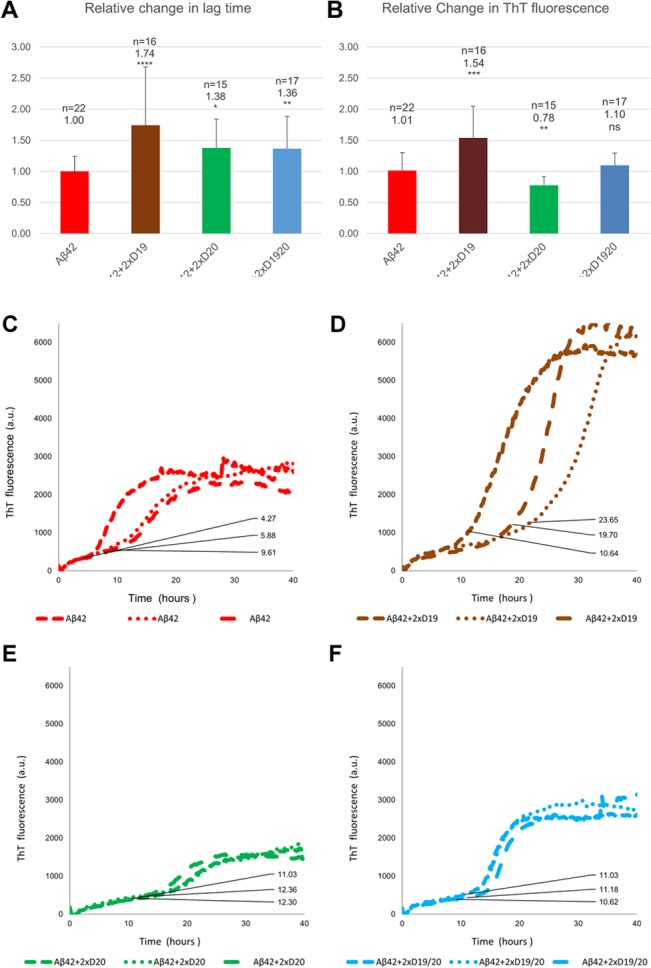
10 μM Aβ 1–42 fibril aggregation in the presence of 20 μM peptides over 40 hours. (A-B) Data from eight experiments (total n = 22 for Aβ 1–42) is shown. Error bars = SD. * p < 0.05, ** P < 0.01, *** p < 0.001, **** p < 0.0001. (A) Average changes in lag time relative to Aβ 1–42 alone (normalized to 1). (B) Average changes in ThT fluorescence relative to Aβ 1–42 alone (normalized to 1). (C-F) A representative kinetic experiment showing the inherent variability of Aβ 1–42 aggregation in the absence (C) or presence of D19 (D), D20 (E) or D19/20 (F) in replicate wells (n = 3). Despite this variability, the lag phases are always prolonged in the presence of D-peptide.

Because the kinetics of Aβ 1–42 aggregation are notoriously variable[36], eight separate experiments with internal replicates of up to five were performed (Fig 2). Total n values for Aβ 1–42 alone or plus D19, D20 and D19/20 were 22, 16, 15 and 17 respectively (Fig 2, S1 Table). Changes in lag phase or final ThT fluorescence were normalized to the average lag phase or average change in fluorescence of control Aβ 1–42 for each experiment (Fig 2A and 2B) (see methods for details of calculation). Over all the experiments, lag phases for 10 μM Aβ 1–42 controls ranged from 2.6 to 9.6 hours, but lag phases were consistently prolonged by addition of peptides in all experiments (an example of a typical experiment with internal replicates is shown in Fig 2C). The normalized average lag phase for Aβ 1–42 controls was 1.00 ± 0.24. Co-incubation with 20 μM peptide (a 2:1 ratio of peptide:Aβ 1–42) led to significantly prolonged lag phases for all the peptides, as seen with the dose response curves at the same ratio but half the concentration (S3 Fig). D19 was the longest at 1.74 ± 0.94 fold higher; increases of 1.36 ± 0.52 and 1.38 ± 0.46 fold were observed with D19/20 and D20 respectively (Fig 2A, S1 Table). All aggregation reactions were performed for 40 hours minimum.

In addition to prolonging lag phase, each peptide had a particular and consistent effect on the change in ThT fluorescence (Fig 2B). Co-incubation of Aβ 1–42 with D19 generated aggregates with significantly larger changes in ThT fluorescence values than Aβ 1–42 alone (1.54 ± 0.51 vs 1.01 ± 0.29). In contrast, co-incubation with D20 produced kinetics with significantly smaller changes (0.78 ± 0.14 fold less) and co-incubation with D19/20 did not affect the change in ThT value (1.10 ± 0.20). It is important to note that different fibril structures can differentially bind ThT[37]; therefore the change in ThT may correlate with the number of fibrils or reflect a change in morphology of the fibrils.

### Morphological analysis of fibrils

**Fig 3:**
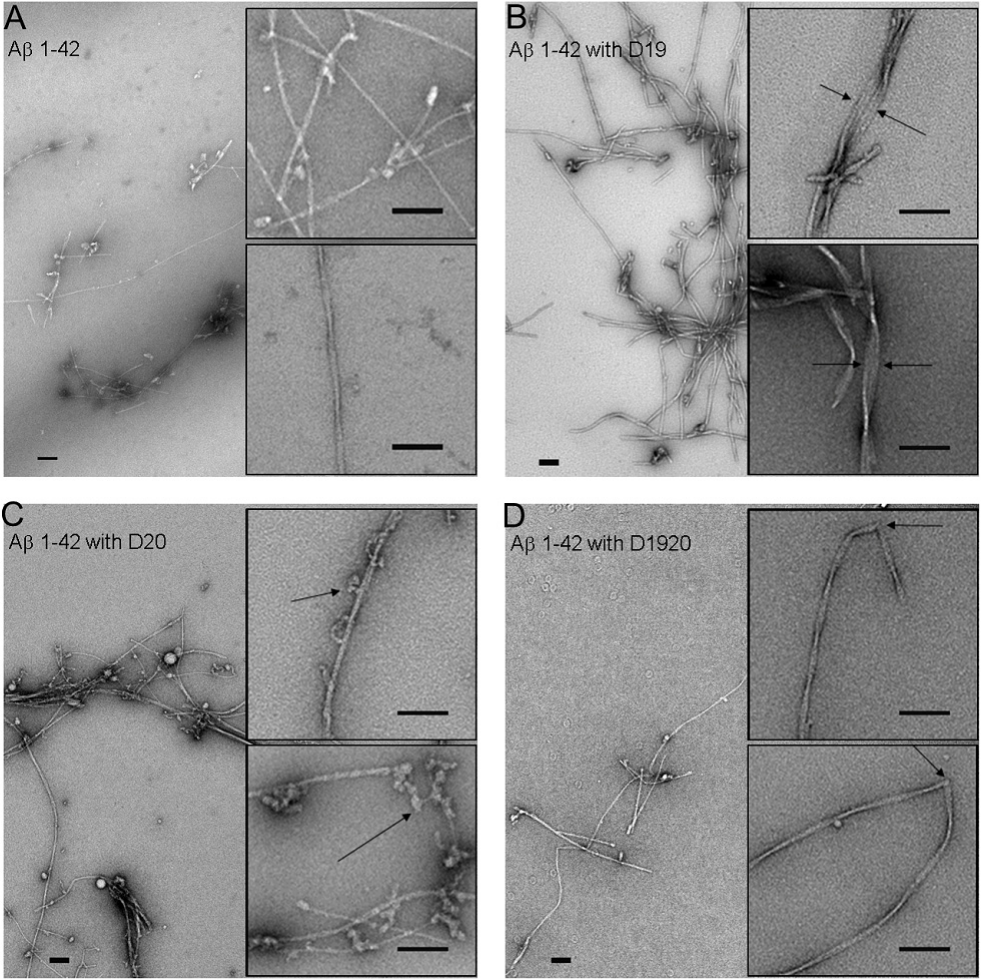
Transmission electron micrographs of end-products of 48 hour Aβ 1–42 fibril (37°C) reactions in the presence or absence of peptides. (A) Aβ 1–42 fibrils alone. (B) Aβ 1–42 fibrils after co-incubation with D19. A number of laterally associated fibrils are seen (arrow). (C) Aβ 1–42 fibrils after co-incubation with D20. Frequent oligomeric structures are associated along the length of many fibrils (arrow). (D) Aβ 1–42 fibrils after co-incubation with D19/20. Many fibrils had kinks or bends (arrow). Scale bars are 100 nm.

To confirm that the ThT positive aggregates produced in the co-incubation kinetic assays were fibrillar, and to further determine whether they had any visible morphological differences that might explain the different ThT values, we examined the end-products of 48 hour aggregation reactions by EM. Reactions with ratios of 3:1 were examined, as we predicted that these would the greatest morphological differences, given that these also had the greatest alterations in lag phase and ThT levels in our original dose curve treatments (S3 Fig). Electron micrographs revealed fibrils in each group (Fig 3). Many fibrils showed periodic twisting, as expected for amyloid fibrils[38], and we compared fibril characteristics (periodicity and minimum and maximum widths) from each group (Table 1). Fibrils from D19/20 reactions had significantly higher periodicities (shorter distances between turns) whereas those from D20 reactions had lower periodicities (longer distances between turns), as compared with Aβ 1–42 control fibrils. With respect to fibril widths, fibrils generated in the presence of D19 were significantly wider at both their maximum (17.6 +/- 4.9 nm) and minimum (8.4 +/- 1.8 nm), as compared to Aβ 1–42 fibrils alone (maximum width 14.0+/- 2.4 nm; minimum width 6.8 +/- 2.0 nm).

**Table 1:** Properties of Aβ 1–42 fibrils generated in the presence or absence of peptides.

	Periodicity(nm)				width (nm) at max				width (nm) at min			
	Average	S.D.	n		Average	S.D.	n		Average	S.D.	n	
Aβ42	137.1	29.9	25		14.0	2.4	26		6.8	2.0	23	
Aβ42+D19	153.0	46.4	14		17.6	4.9	20	*	8.4	1.8	18	*
Aβ42+D20	171.1	26.0	48	*	14.6	2.3	49		7.5	1.3	50	
Aβ42+D19/20	110.7	20.4	11	*	13.6	2.4	14		5.7	1.1	7	

* p < 0.05.

Several other qualitative features were noted by EM. In the presence of D20, fibrils had bead-like oligomeric structures coating the mature fibrils (Fig 3C). By contrast, with D19, few if any oligomeric structures were observed. In the presence of D19/20, fibrils showed an increased tendency to form kinks which were 500–600 nm apart (Fig 3D).

### Morphological analysis of oligomers

**Fig 4:**
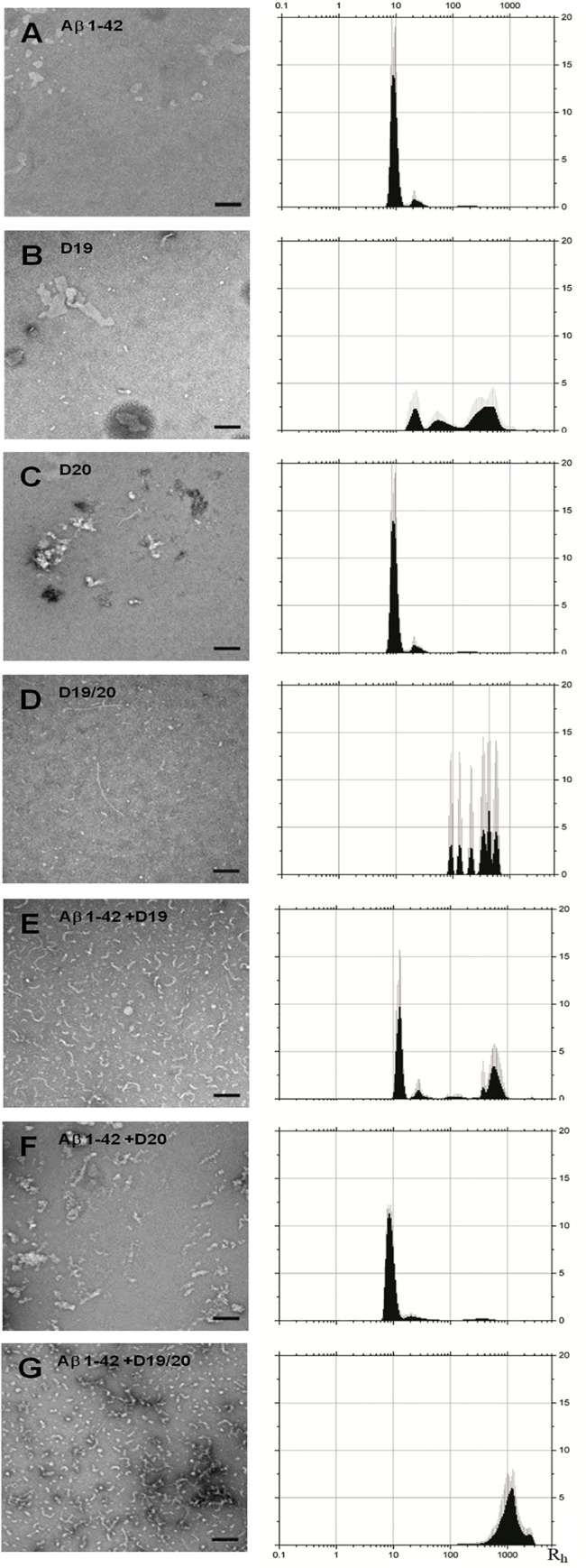
Electron microscopy and dynamic light scattering analysis of end-products of 24 hour oligomer (4°C) preparations in the presence or absence of peptides. (A) Aβ 1–42, (B) D19, (C) D20, (D) D19/20, (E) Aβ 1–42 + D19, (F) Aβ 1–42 + D20 and (G) Aβ 1–42 + D19/20. Scale bars are 100 nm. The size (Rh) distribution by mass has been plotted. Averages of ten readings are shown with error bars representing standard deviation.

**Fig 5:**
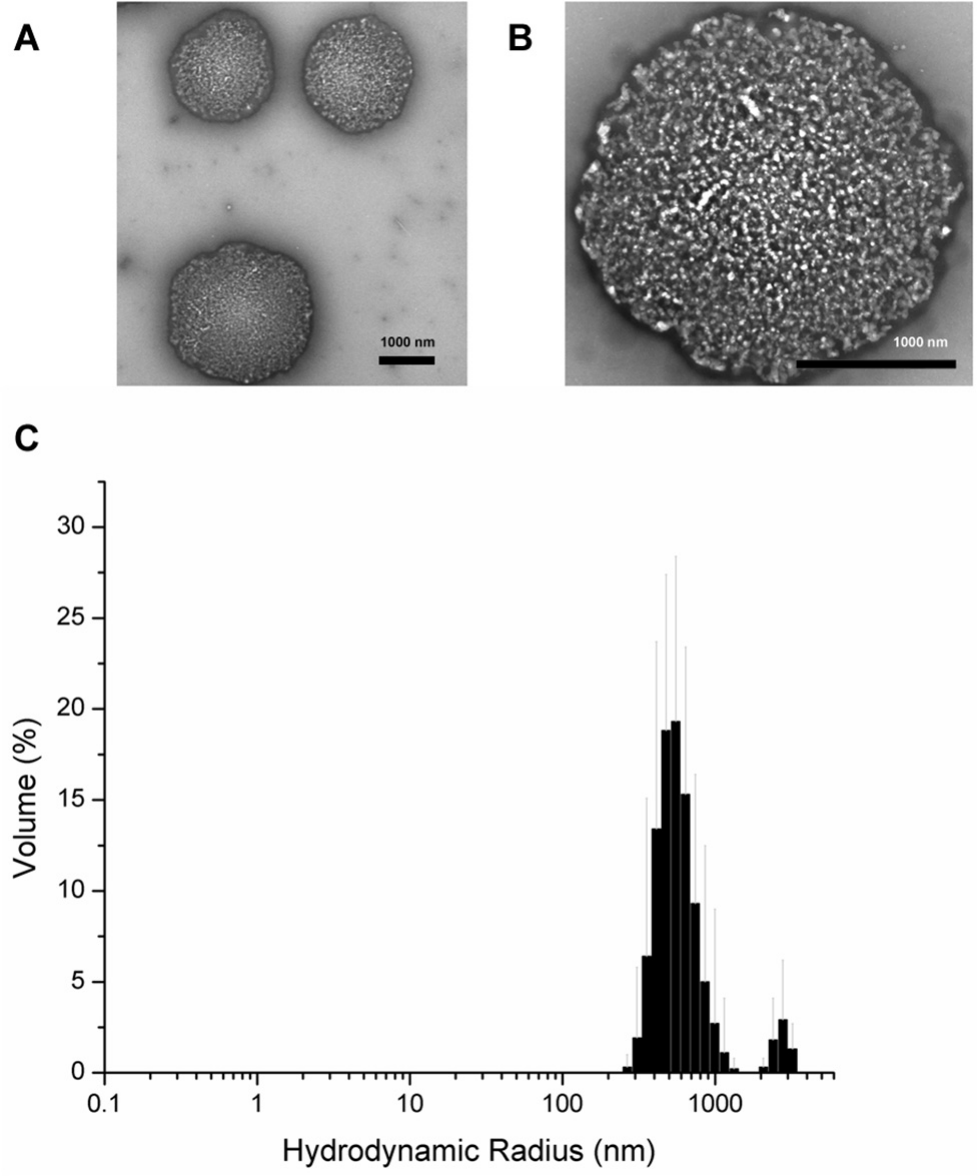
Morphology of D19/20 large spherical aggregates. (A-B) Electron micrographs of occasionally observed large aggregates from D19/20 oligomer incubation reactions. Scale bar 1000 nm. (C) Rh distribution of aggregates. Average of twelve readings shown. Error bars represent standard deviation.

Because oligomers of Aβ are the more relevant toxic species compared to fibrils[39–40], we tested whether co-incubation of our peptides with Aβ would affect oligomerization. To form oligomers, we used a standard method for producing Aβ oligomers, which uses the same buffer conditions as for fibril formation but reactions are incubated at 4°C (instead of 37°C) for at least 24 hours[41]. We generated oligomers from peptides alone or Aβ 1–42 in the presence of 2:1 peptide:Aβ 1–42. Using dynamic light scattering (DLS) in batch mode plus EM, we determined the size distributions of oligomeric products at the end of their 24 hour 4°C incubation (Fig 4). Averages of ten readings are shown, with standard deviation indicated by error bars. The distribution of all particles is shown, with area under the curve totalling 100%. Aβ 1–42 samples formed aggregates of less than 10 nm radius which were not readily resolved by EM (Fig 4A). D20 oligomers had a similar profile (Fig 4C). In contrast, oligomer reactions containing only D19 or D19/20 produced greater size distributions and overall larger aggregates, some of which had Rh values greater than 600 nm. By EM, all the samples contained primarily amorphous aggregates (Fig 4B and 4C), with occasional small oligomeric material seen in D19/20 (Fig 4D). Interestingly, lower magnification images of D19/20 oligomer samples revealed occasional large 1 μm spheres of what appeared to be aggregates of amorphous material (Fig 5). None of the oligomer products were ThT fluorescent (data not shown).

Oligomers containing Aβ 1–42 plus D20 (Fig 4F) largely resembled those of Aβ 1–42 alone with respect to size distribution, but some amorphous aggregates could be detected by EM. Oligomers containing Aβ 1–42 plus D19 had a population of aggregates with Rh values similar to those from Aβ 1–42 alone (~ 10 nm) in addition to populations of larger aggregates as seen for D19 alone. Interestingly, for oligomers of Aβ 1–42 plus D20, no smaller populations were detected, only large aggregates, some of which had Rh values exceeding 1100 nm. Although the 1 μm spherical aggregates seen in the D19/20 alone samples were not observed in the Aβ 1–42 plus D19/20 mixed sample, there were numerous amorphous aggregates which resembled those seen in the larger spherical aggregates, suggesting the larger aggregates may have been broken up by pipetting prior to EM grid preparation.

### Toxicity

**Fig 6:**
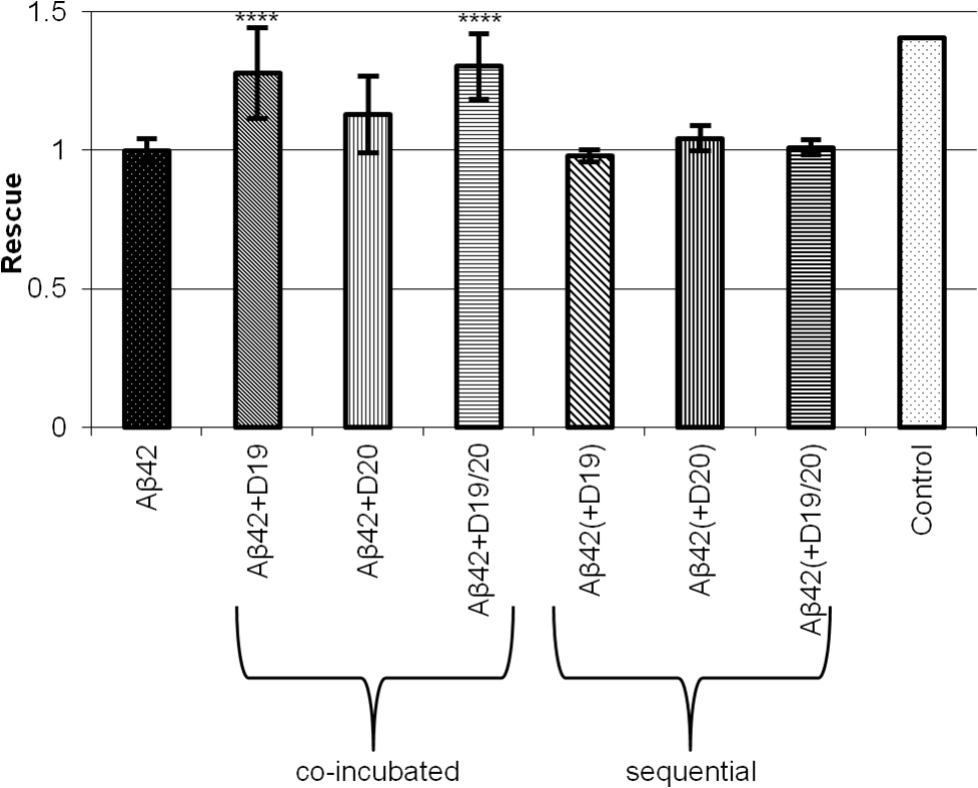
Rescue of cell viability after treatment with Aβ 1–42 oligomers co-incubated with D19, D20 or D19/20, as measured by MTS assay in primary cortical neurons. Cell toxicity was partially rescued when cells were treated with oligomer reactions of Aβ 1–42 co-incubated with peptide (Aβ42+peptide). No rescue was observed if cells were treated sequentially with Aβ 1–42 oligomers and then peptide oligomers (Aβ 1-42(+peptide)), without prior co-incubation. Values shown are normalized to Aβ 1–42 toxicity levels. n ≥ 6. ** p < 0.01.

To determine whether our peptides influenced the toxicity of Aβ 1–42 oligomers, we used a formazan-based viability assay (MTS) on rat primary cortical neuronal cultures after treatment with oligomers for 48 hours. Oligomers were prepared as described above, by 24 hour incubation at 4°C. Cultures treated with 15 μM Aβ 1–42 oligomer controls had cell viabilities ranging from 62 ± 2% to 83 ± 4%; control reactions treated with 15 μM peptide oligomers alone were not toxic to cells (S5 Fig). Treating cultures with Aβ 1–42 oligomers generated by co-incubation with D19 or D19/20 at 4°C for 24 hours (final concentration of 15 μM Aβ 1–42 and 30 μM peptide oligomer), improved the viability of cells relative to those treated with Aβ 1–42 oligomers only (D19 increased viability by 1.28 ± 0.16 fold, D19/20 by 1.30 ± 0.12 fold). Co-incubation with D20 led to a 1.13 ± 0.13 increase in cell viability, but this was not statistically significant (Fig 6).

This protective effect was only evident when peptides were co-incubated with Aβ 1–42 during oligomer formation; when oligomeric or monomeric peptides (30 μM) were added to the cells sequentially, after treatment with Aβ 1–42 oligomers (15 μM) formed in isolation, no rescue was observed (Fig 6).

Given that Aβ 1–42 fibrils were still able to form in the presence of the peptides, we tested whether the toxicity of Aβ 1–42 aggregates was altered, even though Aβ 1–42 fibrils are generally less toxic to cells. Treating N2a or SH-SY5Y cell cultures for 24 or 48 hours, we confirmed that the overall toxicity of fibrils was less than the oligomers. However, a similar protective effect was again observed after treatment with Aβ 1–42 fibrils generated in the presence of peptides (S6 Fig).

### Dynamic light scattering

**Fig 7:**
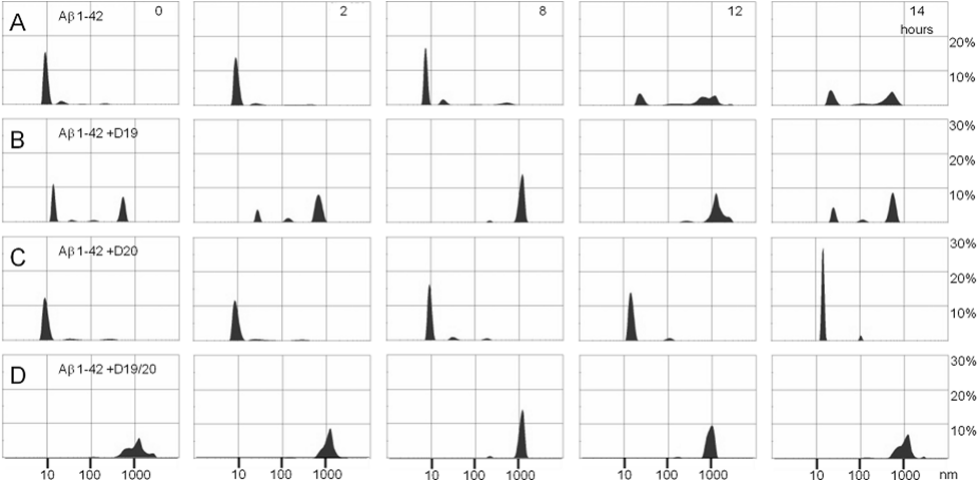
Dynamic light scattering during fibrillization. Size (Rh) distribution by mass for Aβ 1–42 alone (A) and in presence of D19 (B), D20 (C) and D19/20 (D) observed at 0, 2, 8, 12 and 14 hours under fibril forming conditions at 37°C. The starting material was first oligomerized by incubation at 4°C for 24 hours.

Given that oligomers readily form fibrils when placed at 37°C, we wanted to establish whether a transient species, on or off pathway to fibril formation, might correlate with the toxicity seen in cell culture exposed to oligomers. We therefore created oligomers at 4°C in 2:1 ratios (peptide: Aβ 1–42), determined their initial size distribution (Fig 4) and then followed their size distribution during subsequent incubation at 37°C (Fig 7) to partially mimic the environment under which oligomers exist once put into cell culture, albeit without the complexities of cell processing or co-factor interaction. Aβ 1–42 oligomers retained their size distribution until 8 hours, at which point larger populations with Rh values approaching 1000 nm started to appear (Fig 7A). This timing correlates well with the lag phase we usually see for Aβ 1–42 aggregation. For Aβ 1–42 plus D20 (Fig 7C), the profile was similar to that of Aβ 1–42 but without a significant population of aggregates with Rh values larger than 100 nm. For Aβ 1–42 plus D19 (Fig 7B), an additional population was seen initially, with Rh values of approximately 500 nm; this larger population did not dramatically change over the course of incubation. Aβ 1–42 plus D19/20 (Fig 7D) started as very large aggregates and was the only sample that did not have any detectable small aggregates at the start of the experiment.

## Discussion

There is considerable effort being put into the development of therapeutics and early diagnostic tools for AD, as therapeutic success will likely be improved if treatments can be administered early. The earliest event in AD pathogenesis is thought to be Aβ accumulation. As such, this is a prime target for early diagnosis and therapeutic intervention. For the present study, we chose to sterically modify the self-aggregating 14–23 segment of Aβ in order to increase the specificity and strength of peptide binding and its incorporation into growing Aβ fibrils, given that residues 14–23 comprise the accessible exterior of the amyloid core in fibrils of Aβ 1–42[14] (Fig 1). Then, by using D-amino acid substitution to target phenylalanine residues 19 and 20 within this peptide, we were able to alter both the aggregation profiles and toxicity of Aβ 1–42 aggregates. Peptides with this ability to both incorporate into an aggregate and reduce toxicity may be amenable to both therapeutic and diagnostic imaging tools.

The only other study to specifically target the single residues 19 or 20 with D amino acid substitution used the hexamer KLVFFA at a 10:1 ratio with Aβ 1–40, where a small decrease in ThT fluorescence of the aggregates was demonstrated[34]. In our study, we used the longer peptide 14–23 in lower ratios with Aβ 1–42 and assessed effects on both fibril and oligomer formation, identifying correlations with toxicity profiles.

Importantly, unlike the L-peptide 14–23, D19, D20 and D19/20 peptides did not form ThT positive aggregates or fibrils, but were able to incorporate into the growing Aβ 1–42 aggregate, likely during nucleation, given the increased lag phases and altered fibril morphologies. This incorporation may have distorted the π stacking arrangement of phenylalanines F19 and F20. In mature Aβ 1–42 fibrils, F19 stacks within the fibril core and is solvent inaccessible while F20 is solvent accessible[14]. Altering the relative relationship of these two aromatic rings with a single D-amino acid substitution at either D19 or D20 was equally effective at delaying aggregation; maintaining the relative relationship through double substitution (D19/20) was less effective. This supports the hypothesis that steric hindrance around these residues is influential in the aggregation process[35]. Interestingly, Aβ 1–42 fibrils generated in the presence of D19 were significantly thicker than Aβ 1–42 fibrils alone, suggesting lateral assembly may have occurred, as has been reported for recombinant prion protein fibrils[42]. Why these fibrils would be more prone to this assembly is not clear, but the relocation of the aromatic residues to the exterior of the core may have allowed a lateral π stacking to occur. This lateral assembly was not observed in Aβ 1–42 control fibrils, nor those formed during co-incubation with D20 or D19/20. The accessibility of aromatic residues may have also affected the number of ThT binding sites, with higher ThT fluorescence seen in fibrils generated with D19 and lower levels for those with D20.

Periodicity was significantly higher in D19/20-associated and lower in D20-associated Aβ 1–42 fibrils. This suggests that, by incorporating into the stacking fibril structure, D19/20 imposed a greater degree of twist based on altered π stacking, thus increasing the average periodicity in the fibrils. Conversely, D20 positions a second aromatic residue within the core interior which would sterically increase the size of the fibril core and possibly restrict efficient stacking, leading to a longer turnover distance and decreased periodicity. This is similar to bulge bending in DNA helices, where the mismatch of a single base pair causes a kink or bulge in the helix. Interestingly, in DNA, these bulges serve as recognition sites for ligands[43–44], raising the possibility that a similar kink in Aβ 1–42 fibrils could produce an accessible binding site to facilitate the clearance of Aβ deposits.

Perhaps more important than structural differences induced in Aβ 1–42 fibrils were our findings that the D-enantiomers were non-toxic, did not aggregate on their own and actually reduced the toxicity of newly forming Aβ 1–42 aggregates. When we look for features that correlate with the reduction in toxicity, we find that changes affecting fibrils, such as changes in final ThT fluorescence and lag phase prolongation, do not correlate with changes in toxicity. All peptides caused a prolonged Aβ 1–42 lag phase, with D19 and D20 prolonging lag phase similarly, but only D19 and D19/20 significantly improved cell viability. In addition, each peptide had a different effect on final fibril ThT values.

**Fig 8:**
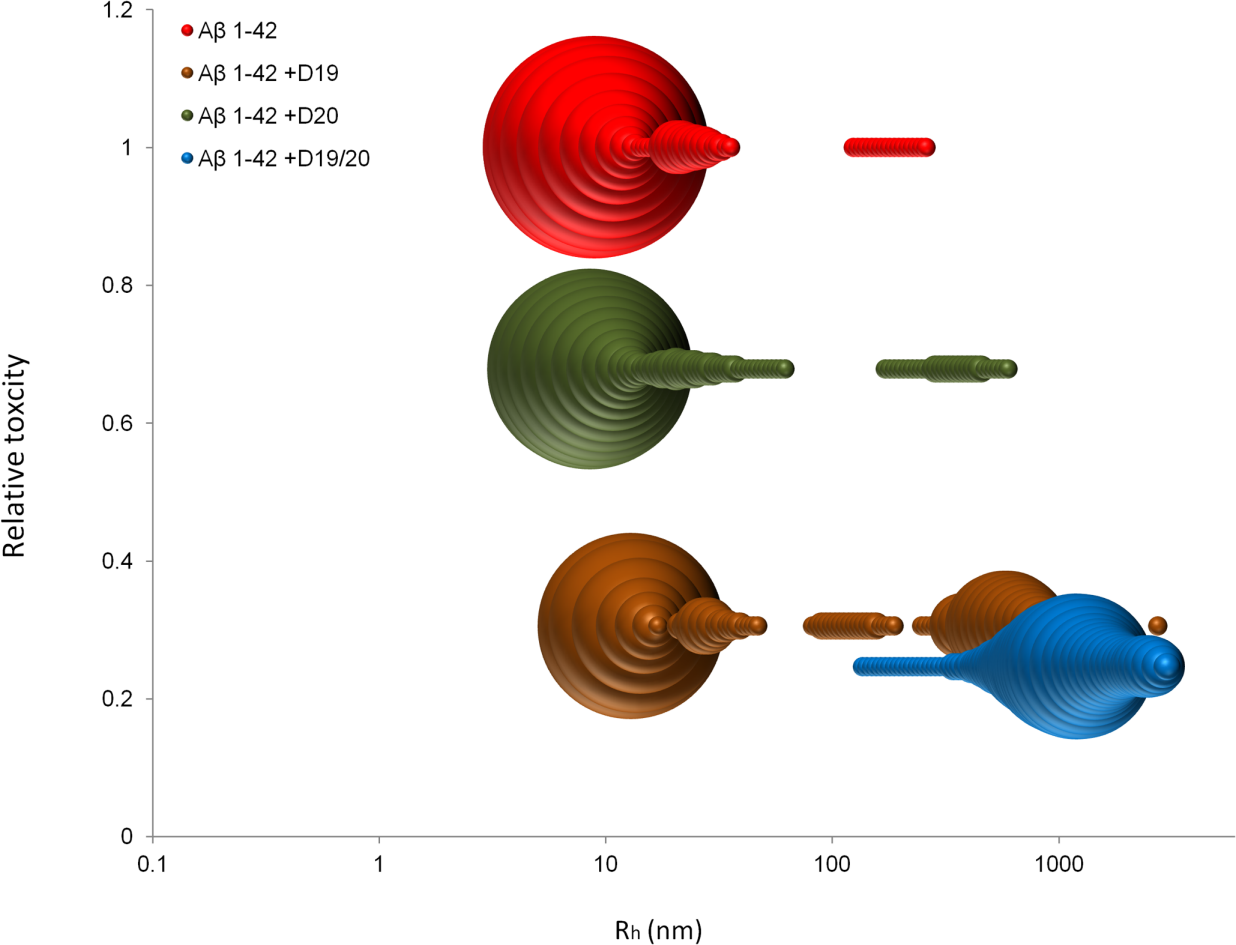
Aβ 1–42 toxicity correlated with hydrodynamic radii of oligomers. A bubble plot correlates the size and toxicity observed for Aβ 1–42 oligomers with or without peptide co-incubation at 4°C. The size of each bubble represents the percentage of mass with that particular radius.

Instead, the feature that best correlates with improved cell viability is oligomer size distribution, with larger aggregates being more favourable (Fig 8). When Aβ 1–42 oligomers were formed in the presence of D19 or D19/20, a larger aggregate population was maintained, whereas in those formed with D20, the profile was comparable to Aβ 1–42 over time (Fig 7). Aβ 1–42 oligomers were the most toxic and the majority of the oligomers had Rh values of 10 nm. The presence of this sized aggregate alone was not sufficient to cause toxicity though, as aggregates of a similar Rh were present in large amounts in Aβ 1–42 plus D19 and these preparations were much less toxic. The key difference was the co-existence of larger aggregates, approaching Rh values of 1000 nm. In fact, for AB plus D19/20, only very large aggregates were seen and these samples were the least toxic. Thus, we can postulate that the larger aggregates are less toxic and can protect cells from the smaller toxic oligomers if they are generated in co-incubation conditions. Based on our EM studies of the very large aggregates, we can further propose that D19 and D19/20 are more likely to form large amorphous aggregates of oligomers. Co-incubation with Aβ 1–42 oligomers during formation may lead to sequestration of the Aβ 1–42 oligomers within these larger aggregates, thus limiting their ability to exert any toxic effects on cells. This explains why we only see protection after co-incubation; if the large aggregates are added to cells after the cells have already been exposed to Aβ 1–42 oligomers, sequestration is less efficient and there is no rescue of toxicity. There is precedent for this concept of sequestration, including at the level of the brain where Aβ 1–42 plaques may act as sinks, entrapping Aβ 1–42 oligomers and thereby reducing toxicity[45].

While this sequestration of more toxic oligomers into larger aggregates can explain a number of our experimental results, it is also probable that other factors are involved. For example, D20 forms oligomers comparable in size to Aβ 1–42, however the toxicity profile for D20 oligomers is lower than for Aβ 1–42 oligomers, and is actually equal to those of D19 and D19/20, both of which form larger oligomers. Therefore a factor other than size must be affecting toxicity here. In addition, while co-incubation of Aβ 1–42 with D19 or D19/20 produced less toxic products, some with significantly larger sizes than the other reactions, Aβ 1–42 plus D19 still produced a majority of oligomers with sizes comparable to Aβ 1–42. Thus it is possible that, rather than size, the actual surface structure of the D-peptide and its co-incubated aggregates are different and it is the exposure of cells to an alternate oligomer binding site that reduces the toxic effect, either through reduced binding or binding which is inconsequential. A dominant inhibitory effect is unlikely, as the sequential addition of Aβ 1–42 followed by D-peptide oligomer was not able to rescue the cells. An altered surface structure could also secondarily affect aggregation by thermodynamically favouring certain sizes. While we cannot prove this model within the scope of the current work, it provides a rationale to further study these oligomers at the molecular level.

## Conclusions

This study establishes that D-enantiomers of phenylalanine at position 19 or 19 and 20 in Aβ 14–23 peptides can modulate Aβ 1–42 aggregation in favour of non-toxic oligomerization, possibly via alteration of oligomer surface binding sites and / or sequestration of Aβ 1–42 into large amorphous aggregates. This is without the formation of toxic aggregates themselves. These peptides are small (~1.2 kDa) and highly soluble in water, therefore should be able to cross the blood brain barrier [46]. Because they are incorporated into the Aβ 1–42 aggregates, they have potential not only as therapeutic agents, but could be used as a basis for the design of ^19^F labelled diagnostics.

## Methods

### Ethics statement

This study was carried out in strict accordance with the recommendations in the Canadian Council on Animal Care, as approved by the Animal Care and Use Committee (ACUC) of the University of Alberta (Study ID AUP00000271). 18 day-old embryos of timed pregnant BALB/c mice were anesthetized with halothane prior to decapitation.

### Peptide synthesis and purification

The peptides were synthesised by using standard Fmoc-based solid phase synthesis on a Liberty-1 microwave peptide synthesizer (CEM) using amide coupling at the C-terminus with Fmoc-Wang-resin. Peptides were cleaved from the resin by treatment with trifluoroacetic acid (TFA, 94%) in the presence of triisopropylsilane (2.5%) and water (2.5%), and precipitated with ice cold diethyl ether. The precipitated peptides were dissolved using acetonitrile/water and solubilised peptides were purified using a reverse phase (vydac) C18 column on HPLC (Gilson) and analysed by MALDI-TOF mass spectrometry. Purified peptides were freeze-dried and dissolved in deionised water prior to experiments.

### Aβ aggregation kinetics assay

Aβ 1–42 (American Peptides) was stored at -80°C and equilibrated at room temperature for 30 minutes before resuspending in 1,1,1,3,3,3-Hexafluoro-2-Propanol (HFIP) to obtain a 1 mM solution. The vial was vortexed to obtain a clear solution and incubated at room temperature for 2 hours to allow monomerization. The Aβ 1-42/HFIP solution was divided into 0.2 mg Aβ 1–42 aliquots in 0.5 mL polypropylene tubes. The solution was concentrated using a SpeedVac centrifuge (800g, room temperature) until a clear peptide film was observed. Vials were then sealed and stored at -20°C and were used within a month from preparation. A fresh vial was used to prepare a 5 mM Aβ 1–42 solution in DMSO prior to each experiment, and was subsequently diluted to 100 μM Aβ 1–42 with deionised water and sonicated for 5 minutes [41]. The Aβ 1–42 aggregation reactions were performed by incubating full length Aβ peptide (1–42) with or without the synthesized peptides in 10 mM sodium phosphate, 50 mM sodium chloride, pH 7.4 at 37°C and aggregation was monitored by Thioflavin T (ThT) fluorescence at 482 nm (Spectramax M5 spectrophotometer). The concentration of Aβ 1–42 was 10 π M for the kinetic analyses, 5 μM for the dose response curve. The data points were recorded every 15 minutes with 30 seconds shaking prior to each reading. The Aβ 1–42 oligomers were prepared in a similar manner by incubating at 4°C[41].

Raw data was fitted using the following equation [47] to determine the lag time based on ThT fluorescence. The *y*_*0*_ and *y*_*f*_ represent initial and final ThT fluorescence respectively. The apparent rate constant (k_app_) can be derived by *1/τ* and lag time (L) is calculated using *t*_*0*_ *-2τ*.

$$\text{y}={\text{y}}_{0}+[{\text{y}}_{\text{f}}/\;(1+{\text{e}}^{\;-(\text{t}-{\text{t}}_{0})/\tau })]$$

The change in lag phase for co-incubated Aβ 1–42 and peptide was calculated relative to the lag time for Aβ 1–42 for each experiment using the formula:

$$\text{Relative lag phase}=1+\left[\left({\text{L}}_{\text{peptide}}-{\text{L}}_{\text{A}\beta 42}\right)/\;{\text{L}}_{\text{A}\beta 42}\right]$$

where L_peptide_ is the lag phase for each well from a given experiment and L_Aβ42_ is the average lag phase from all Aβ samples for that given experiment.

The change in ThT fluorescence for co-incubated Aβ 1–42 and peptide was calculated relative to the change in ThT fluorescence for Aβ 1–42 for each experiment using the formula:

$$\text{Relative change in ThT fluorescence}=1+\left[\left(\Delta {\text{F}}_{\text{peptide}}-\Delta {\text{F}}_{\text{A}\beta 42}\right)/\Delta {\text{F}}_{\text{A}\beta 42}\right]$$

where ΔF_peptide_ is the difference between initial and final fluorescence for each well from a given experiment and ΔF_Aβ42_ is the average difference between initial and final fluorescence from all Aβ samples for that given experiment.

The statistical significance (P<0.05) was calculated using a two-tailed t test using Graphpad Prism software.

### Cell cultures

Mouse neuroblastoma cells (N2a) (ATCC CCL-131) or human neuroblastoma cells (SH-SY5Y) (ATCC CRL-2266) were cultured with DMEM with 5% FBS and PenStrep (Gibco). Cells were plated at 40% confluence in a 96-well plate and grown for at least 48 hours prior to the experiment. Mouse primary cortical neurons were prepared from 18 day-old embryos of timed pregnant BALB/c mice[48–50]. The cell suspension was filtered through a cell strainer and then plated on 96-well plates. The cultures were grown at 37°C in a 5% CO_2_ humidified atmosphere in Neurobasal medium supplemented with B27, 50 μM L-glutamine, 15 mm HEPES, 10 units/mL penicillin, 10 mg/mL streptomycin, and 1% FBS. The medium was replaced 1 day later without FBS, and all experiments were performed on day 6/7 after plating.

### Toxicity

For toxicity experiments, cultures were treated for 48 hours with or without (as control) different concentrations (2–20 μM) of Aβ 1–42 and D19, D20 and D19/20 peptides. Cytotoxicity was evaluated by the 3-[(4,5-dimethylthiazol-2-yl)-5,3-carboxymethoxyphenyl]-2-(4-sulfophenyl)-2H tetrazolium, inner salt (MTS) reduction assay (Promega). Cells were incubated with MTS solution (2 mg/mL) for 2 hours at 37°C in a 5% CO_2_ incubator, then read at 490 nm using a Spectramax M5 spectrophotometer. All data are expressed as the mean ± SD. Statistical significance was calculated using one-way ANOVA followed by Dunnett's multiple comparisons test. P values lower than 0.05 were considered significant.

### Electron microscopy (EM)

Five μL aliquots of the peptide reaction solutions were placed on 300 mesh carbon-coated copper grids for 2 minutes followed by two washes with water. After removal of excess liquid, samples were negatively stained using 2% uranyl acetate. The dried samples were examined in a Hitachi H-7650 transmission electron microscope at 80 or 60 kV.

### Light scattering

Dynamic light scattering (DLS) experiments were performed with a Malvern Zetasizer-Nano S. A 633 nm wavelength HeNe laser was used to detect backscattered light at a fixed angle of 173°. The software (DTS v6.20) provided both the mean size and polydispersity by cumulants analysis. We assumed the solution viscosity and refractive index (1.33) to be that of water for calculation purposes. The cell holder was maintained at 20°C for the first measurement of 4°C generated oligomers. Data were collected using a 3 mm x 3 mm quartz cuvette filled with 45 μL of sample and 45 μL of mineral oil on top to avoid evaporation. The data was collected without attenuation and a minimum number of 10 consecutive runs of 10 seconds each was averaged to obtain the autocorrelation function. Particle size was calculated by the manufacturer’s software through the Stokes-Einstein equation assuming spherical shapes of the particles. DLS is extremely sensitive to the presence of large aggregates.

## Supporting Information

S1 Fig Fibril characteristics and toxicity of Aβ 14–23 peptide (a control peptide composed of all L amino acids). ThT fluorescence kinetics and EM of samples from the starting time of the reaction are shown for Aβ 14–23 (A and B), Aβ 1–42 alone (C and D), and Aβ 1–42 in the presence of Aβ 14–23 (E and F). (G) SHSY-5Y cell viability after 48 hours of exposure to Aβ 1–42 fibrils, Aβ 14–23 fibrils, or Aβ 1–42 fibrils formed while co-incubated with Aβ 14–23 (n = 3, * p < 0.05m *** p < 0.001, **** p < 0.0001).
(PDF)

S2 Fig Thioflavin T fluorescence kinetic curves for peptides under fibril forming conditions. (PDF)

S3 Fig Dose-dependent thioflavin T fluorescence kinetic curves for Aβ 1–42 plus peptides under fibril forming conditions. (A-C) Reactions were carried out in the presence of 5 μM Aβ 1–42 plus variable ratios of the peptides. (D) A linear increase in lag time is seen with increasing peptide concentration.
(PDF)

S4 Fig Thioflavin T fluorescence kinetic curves of Aβ 1–42 in the presence of D20 peptide. (A) Aβ 1–42 (10 μM) with 1, 2 and 4 fold molar excess of D20 peptide. (B) Aβ 1–42 (2.5 μM) with 8 and 32 fold molar excess of D20 peptide.
(PDF)

S5 Fig Cell viability after exposure to oligomer preparations of D19, D20 and D19/20, as compared to Aβ 1–42 oligomer exposure. Primary cortical neurons were treated with oligomeric preparations of the peptides for 48 hours. The cell viability (by MTS assay) after exposure to Aβ 1–42 oligomers was averaged and normalized to 1 for all experiments. Viabilities for each peptide were normalized to the control Aβ 1–42 values for all experiments and then averaged across experiments. n ≥ 6, *** p < 0.001, **** p < 0.0001.
(PDF)

S6 Fig Rescue from Aβ 1–42 fibril-induced cell toxicity as observed in SH-SY5Y cells. Cells were exposed for 48 hours to Aβ 1–42 fibrils, peptides incubated in fibril forming conditions, or Aβ 1–42 fibrils formed through co-incubation with peptides (Aβ 1–42 + peptide). Significant rescue was observed for all co-incubated samples, compared to Aβ 1–42 fibrils alone. n ≥ 6, *** p < 0.001, **** p < 0.0001.
(PDF)

S1 Table Summary of relative increases in the lag times seen across all experiments. (PDF)

## References

[1] Hardy J. The amyloid hypothesis for Alzheimer's disease: a critical reappraisal. J Neurochem. 2009;110(4):1129–34. Epub 2009/05/22. doi: JNC6181 [pii] 10.1111/j.1471-4159.2009.06181.x . 19457065

[2] Hardy J. Alzheimer's disease: the amyloid cascade hypothesis: an update and reappraisal. J Alzheimers Dis. 2006;9(3 Suppl):151–3. Epub 2006/08/18. . 16914853 10.3233/jad-2006-9s317

[3] Hardy J. Testing times for the "amyloid cascade hypothesis". Neurobiol Aging. 2002;23(6):1073–4. Epub 2002/12/10. doi: S0197458002000428 [pii]. . 12470803 10.1016/s0197-4580(02)00042-8

[4] Hardy J, Bogdanovic N, Winblad B, Portelius E, Andreasen N, Cedazo-Minguez A, et al Pathways to Alzheimer's disease. Journal of internal medicine. 2014;275(3):296–303. 10.1111/joim.12192 . 24749173

[5] Hardy J, Selkoe DJ. The amyloid hypothesis of Alzheimer's disease: progress and problems on the road to therapeutics. Science. 2002;297(5580):353–6. 10.1126/science.1072994 . 12130773

[6] Hardy JA, Higgins GA. Alzheimer's disease: the amyloid cascade hypothesis. Science. 1992;256(5054):184–5. . 1566067 10.1126/science.1566067

[7] Tomita T. Secretase inhibitors and modulators for Alzheimer's disease treatment. Expert Rev Neurother. 2009;9(5):661–79. Epub 2009/05/01. 10.1586/ern.09.24 . 19402777

[8] Schenk D, Barbour R, Dunn W, Gordon G, Grajeda H, Guido T, et al Immunization with amyloid-beta attenuates Alzheimer-disease-like pathology in the PDAPP mouse. Nature. 1999;400(6740):173–7. Epub 1999/07/17. 10.1038/22124 . 10408445

[9] Haass C, Selkoe DJ. Soluble protein oligomers in neurodegeneration: lessons from the Alzheimer's amyloid beta-peptide. Nat Rev Mol Cell Biol. 2007;8(2):101–12. Epub 2007/01/25. nrm2101 [pii] 10.1038/nrm2101 . 17245412

[10] Tjernberg LO, Callaway DJ, Tjernberg A, Hahne S, Lilliehook C, Terenius L, et al A molecular model of Alzheimer amyloid beta-peptide fibril formation. J Biol Chem. 1999;274(18):12619–25. Epub 1999/04/23. . 10212241 10.1074/jbc.274.18.12619

[11] Bu Z, Shi Y, Callaway DJ, Tycko R. Molecular alignment within beta-sheets in Abeta(14–23) fibrils: solid-state NMR experiments and theoretical predictions. Biophys J. 2007;92(2):594–602. Epub 2006/10/24. S0006-3495(07)70860-3 [pii] 10.1529/biophysj.106.091017 17056725 PMC1751388

[12] Ahmed M, Davis J, Aucoin D, Sato T, Ahuja S, Aimoto S, et al Structural conversion of neurotoxic amyloid-beta(1–42) oligomers to fibrils. Nat Struct Mol Biol. 2010;17(5):561–7. Epub 2010/04/13. nsmb.1799 [pii] 10.1038/nsmb.1799 20383142 PMC2922021

[13] Fawzi NL, Ying J, Ghirlando R, Torchia DA, Clore GM. Atomic-resolution dynamics on the surface of amyloid-beta protofibrils probed by solution NMR. Nature. 2011;480(7376):268–72. Epub 2011/11/01. nature10577 [pii] 10.1038/nature10577 22037310 PMC3237923

[14] Luhrs T, Ritter C, Adrian M, Riek-Loher D, Bohrmann B, Dobeli H, et al 3D structure of Alzheimer's amyloid-beta(1–42) fibrils. Proc Natl Acad Sci U S A. 2005;102(48):17342–7. Epub 2005/11/19. 0506723102 [pii] 10.1073/pnas.0506723102 16293696 PMC1297669

[15] Lu JX, Qiang W, Yau WM, Schwieters CD, Meredith SC, Tycko R. Molecular structure of beta-amyloid fibrils in Alzheimer's disease brain tissue. Cell. 2013;154(6):1257–68. Epub 2013/09/17. S0092-8674(13)01029-5 [pii] 10.1016/j.cell.2013.08.035 24034249 PMC3814033

[16] Paravastu AK, Qahwash I, Leapman RD, Meredith SC, Tycko R. Seeded growth of beta-amyloid fibrils from Alzheimer's brain-derived fibrils produces a distinct fibril structure. Proc Natl Acad Sci U S A. 2009;106(18):7443–8. Epub 2009/04/21. 0812033106 [pii] 10.1073/pnas.0812033106 19376973 PMC2678625

[17] Paravastu AK, Leapman RD, Yau WM, Tycko R. Molecular structural basis for polymorphism in Alzheimer's beta-amyloid fibrils. Proc Natl Acad Sci U S A. 2008;105(47):18349–54. Epub 2008/11/19. 0806270105 [pii] 10.1073/pnas.0806270105 19015532 PMC2587602

[18] Balbach JJ, Petkova AT, Oyler NA, Antzutkin ON, Gordon DJ, Meredith SC, et al Supramolecular structure in full-length Alzheimer's beta-amyloid fibrils: evidence for a parallel beta-sheet organization from solid-state nuclear magnetic resonance. Biophys J. 2002;83(2):1205–16. Epub 2002/07/19. S0006-3495(02)75244-2 [pii] 10.1016/S0006-3495(02)75244-2 12124300 PMC1302222

[19] Balbach JJ, Ishii Y, Antzutkin ON, Leapman RD, Rizzo NW, Dyda F, et al Amyloid fibril formation by A beta 16–22, a seven-residue fragment of the Alzheimer's beta-amyloid peptide, and structural characterization by solid state NMR. Biochemistry. 2000;39(45):13748–59. Epub 2000/11/15. bi0011330 [pii]. . 11076514 10.1021/bi0011330

[20] Makin OS, Atkins E, Sikorski P, Johansson J, Serpell LC. Molecular basis for amyloid fibril formation and stability. Proc Natl Acad Sci U S A. 2005;102(2):315–20. Epub 2005/01/05. 0406847102 [pii] 10.1073/pnas.0406847102 15630094 PMC544296

[21] Sikorski P, Atkins ED, Serpell LC. Structure and texture of fibrous crystals formed by Alzheimer's abeta(11–25) peptide fragment. Structure. 2003;11(8):915–26. Epub 2003/08/09. S0969212603001497 [pii]. . 12906823 10.1016/s0969-2126(03)00149-7

[22] Pawar AP, Dubay KF, Zurdo J, Chiti F, Vendruscolo M, Dobson CM. Prediction of "aggregation-prone" and "aggregation-susceptible" regions in proteins associated with neurodegenerative diseases. J Mol Biol. 2005;350(2):379–92. Epub 2005/06/01. S0022-2836(05)00426-2 [pii] 10.1016/j.jmb.2005.04.016 . 15925383

[23] Tartaglia GG, Cavalli A, Pellarin R, Caflisch A. The role of aromaticity, exposed surface, and dipole moment in determining protein aggregation rates. Protein Sci. 2004;13(7):1939–41. Epub 2004/06/01. 10.1110/ps.04663504 ps.04663504 [pii]. 15169952 PMC2279921

[24] Inouye H, Gleason KA, Zhang D, Decatur SM, Kirschner DA. Differential effects of Phe19 and Phe20 on fibril formation by amyloidogenic peptide A beta 16–22 (Ac-KLVFFAE-NH2). Proteins. 2010;78(10):2306–21. Epub 2010/06/15. 10.1002/prot.22743 . 20544966

[25] Capone R, Jang H, Kotler SA, Kagan BL, Nussinov R, Lal R. Probing structural features of Alzheimer's amyloid-beta pores in bilayers using site-specific amino acid substitutions. Biochemistry. 2012;51(3):776–85. Epub 2012/01/17. 10.1021/bi2017427 22242635 PMC3265145

[26] Hilbich C, Kisters-Woike B, Reed J, Masters CL, Beyreuther K. Aggregation and secondary structure of synthetic amyloid beta A4 peptides of Alzheimer's disease. J Mol Biol. 1991;218(1):149–63. Epub 1991/03/05. 0022-2836(91)90881-6 [pii]. . 2002499 10.1016/0022-2836(91)90881-6

[27] Chen YR, Huang HB, Lo CJ, Wang CC, Su CL, Liu HT, et al Abeta40(L17A/F19A) mutant diminishes the aggregation and neurotoxicity of Abeta40. Biochem Biophys Res Commun. 2011;405(1):91–5. Epub 2011/01/11. S0006-291X(11)00002-7 [pii] 10.1016/j.bbrc.2010.12.133 . 21216230

[28] Paivio A, Nordling E, Kallberg Y, Thyberg J, Johansson J. Stabilization of discordant helices in amyloid fibril-forming proteins. Protein Sci. 2004;13(5):1251–9. Epub 2004/04/21. 10.1110/ps.03442404 13/5/1251 [pii]. 15096631 PMC2286751

[29] Bernstein SL, Wyttenbach T, Baumketner A, Shea JE, Bitan G, Teplow DB, et al Amyloid beta-protein: monomer structure and early aggregation states of Abeta42 and its Pro19 alloform. J Am Chem Soc. 2005;127(7):2075–84. Epub 2005/02/17. 10.1021/ja044531p . 15713083

[30] Reches M, Gazit E. Controlled patterning of aligned self-assembled peptide nanotubes. Nat Nanotechnol. 2006;1(3):195–200. Epub 2008/07/26. nnano.2006.139 [pii] 10.1038/nnano.2006.139 . 18654186

[31] Soto C, Kindy MS, Baumann M, Frangione B. Inhibition of Alzheimer's amyloidosis by peptides that prevent beta-sheet conformation. Biochem Biophys Res Commun. 1996;226(3):672–80. Epub 1996/09/24. S0006-291X(96)91413-8 [pii] 10.1006/bbrc.1996.1413 . 8831674

[32] Sievers SA, Karanicolas J, Chang HW, Zhao A, Jiang L, Zirafi O, et al Structure-based design of non-natural amino-acid inhibitors of amyloid fibril formation. Nature. 2011;475(7354):96–100. Epub 2011/06/17. nature10154 [pii] 10.1038/nature10154 21677644 PMC4073670

[33] Cruz M, Tusell JM, Grillo-Bosch D, Albericio F, Serratosa J, Rabanal F, et al Inhibition of beta-amyloid toxicity by short peptides containing N-methyl amino acids. J Pept Res. 2004;63(3):324–8. Epub 2004/03/31. 10.1111/j.1399-3011.2004.00156.x JPP156 [pii]. . 15049845

[34] Chalifour RJ, McLaughlin RW, Lavoie L, Morissette C, Tremblay N, Boule M, et al Stereoselective interactions of peptide inhibitors with the beta-amyloid peptide. J Biol Chem. 2003;278(37):34874–81. Epub 2003/07/04. 10.1074/jbc.M212694200 M212694200 [pii]. . 12840031

[35] Frydman-Marom A, Rechter M, Shefler I, Bram Y, Shalev DE, Gazit E. Cognitive-performance recovery of Alzheimer's disease model mice by modulation of early soluble amyloidal assemblies. Angew Chem Int Ed Engl. 2009;48(11):1981–6. Epub 2008/11/28. 10.1002/anie.200802123 . 19035593

[36] Teplow DB. Preparation of amyloid beta-protein for structural and functional studies. Methods Enzymol. 2006;413:20–33. Epub 2006/10/19. S0076-6879(06)13002-5 [pii] 10.1016/S0076-6879(06)13002-5 . 17046389

[37] Lindberg DJ, Wranne MS, Gilbert Gatty M, Westerlund F, Esbjorner EK. Steady-state and time-resolved Thioflavin-T fluorescence can report on morphological differences in amyloid fibrils formed by Abeta(1–40) and Abeta(1–42). Biochem Biophys Res Commun. 2015;458(2):418–23. Epub 2015/02/11. S0006-291X(15)00179-5 [pii] 10.1016/j.bbrc.2015.01.132 . 25660454

[38] Serpell LC. Alzheimer's amyloid fibrils: structure and assembly. Biochim Biophys Acta. 2000;1502(1):16–30. Epub 2000/07/19. S0925-4439(00)00029-6 [pii]. . 10899428 10.1016/s0925-4439(00)00029-6

[39] Hartley DM, Walsh DM, Ye CP, Diehl T, Vasquez S, Vassilev PM, et al Protofibrillar intermediates of amyloid beta-protein induce acute electrophysiological changes and progressive neurotoxicity in cortical neurons. J Neurosci. 1999;19(20):8876–84. Epub 1999/10/12. . 10516307 10.1523/JNEUROSCI.19-20-08876.1999 PMC6782787

[40] Cleary JP, Walsh DM, Hofmeister JJ, Shankar GM, Kuskowski MA, Selkoe DJ, et al Natural oligomers of the amyloid-beta protein specifically disrupt cognitive function. Nat Neurosci. 2005;8(1):79–84. Epub 2004/12/21. nn1372 [pii] 10.1038/nn1372 . 15608634

[41] Stine WB Jr., Dahlgren KN, Krafft GA, LaDu MJ. In vitro characterization of conditions for amyloid-beta peptide oligomerization and fibrillogenesis. J Biol Chem. 2003;278(13):11612–22. Epub 2002/12/25. 10.1074/jbc.M210207200 M210207200 [pii]. . 12499373

[42] Makarava N, Bocharova OV, Salnikov VV, Breydo L, Anderson M, Baskakov IV. Dichotomous versus palm-type mechanisms of lateral assembly of amyloid fibrils. Protein Sci. 2006;15(6):1334–41. Epub 2006/05/30. 15/6/1334 [pii] 10.1110/ps.052013106 16731968 PMC2265092

[43] Gupta S, Gellert M, Yang W. Mechanism of mismatch recognition revealed by human MutSbeta bound to unpaired DNA loops. Nat Struct Mol Biol. 2012;19(1):72–8. Epub 2011/12/20. nsmb.2175 [pii] 10.1038/nsmb.2175 22179786 PMC3252464

[44] Nadai M, Palu G, Palumbo M, Richter SN. Differential targeting of unpaired bases within duplex DNA by the natural compound clerocidin: a valuable tool to dissect DNA secondary structure. PLoS One. 2012;7(12):e52994 Epub 2013/01/04. 10.1371/journal.pone.0052994 PONE-D-12-33669 [pii]. 23285245 PMC3532440

[45] Goure WF, Krafft GA, Jerecic J, Hefti F. Targeting the proper amyloid-beta neuronal toxins: a path forward for Alzheimer's disease immunotherapeutics. Alzheimers Res Ther. 2014;6(4):42 Epub 2014/07/22. 10.1186/alzrt272 alzrt272 [pii]. 25045405 PMC4100318

[46] Liu H, Funke SA, Willbold D. Transport of Alzheimer disease amyloid-beta-binding D-amino acid peptides across an in vitro blood-brain barrier model. Rejuvenation Res. 2010;13(2–3):210–3. Epub 2009/12/04. 10.1089/rej.2009.0926 . 19954305

[47] Nielsen L, Khurana R, Coats A, Frokjaer S, Brange J, Vyas S, et al Effect of environmental factors on the kinetics of insulin fibril formation: elucidation of the molecular mechanism. Biochemistry. 2001;40(20):6036–46. Epub 2001/05/16. bi002555c [pii]. . 11352739 10.1021/bi002555c

[48] Amritraj A, Wang Y, Revett TJ, Vergote D, Westaway D, Kar S. Role of cathepsin D in U18666A-induced neuronal cell death: potential implication in Niemann-Pick type C disease pathogenesis. J Biol Chem. 2013;288(5):3136–52. Epub 2012/12/20. M112.412460 [pii] 10.1074/jbc.M112.412460. 23250759 PMC3561536

[49] Amritraj A, Peake K, Kodam A, Salio C, Merighi A, Vance JE, et al Increased activity and altered subcellular distribution of lysosomal enzymes determine neuronal vulnerability in Niemann-Pick type C1-deficient mice. Am J Pathol. 2009;175(6):2540–56. Epub 2009/11/07. S0002-9440(10)60762-6 [pii] 10.2353/ajpath.2009.081096 19893049 PMC2789601

[50] Zheng WH, Bastianetto S, Mennicken F, Ma W, Kar S. Amyloid beta peptide induces tau phosphorylation and loss of cholinergic neurons in rat primary septal cultures. Neuroscience. 2002;115(1):201–11. Epub 2002/10/29. S0306452202004049 [pii]. . 12401334 10.1016/s0306-4522(02)00404-9

