# Supplementary material for: Structure-Based Peptide Design to Modulate Amyloid Beta Aggregation and Reduce Cytotoxicity
Source: PLoS One. 2015 Jun 12;10(6):e0129087. doi: 10.1371/journal.pone.0129087 (PMC4466325; doi:10.1371/journal.pone.0129087)
Supplement: S1 Fig — ThT fluorescence kinetics and EM of samples from the starting time of the reaction are shown for Aβ 14–23 (A and B), Aβ 1–42 alone (C and D), and Aβ 1–42 in the presence of Aβ 14–23 (E and F). (G) SHSY-5Y cell viability after 48 hours of exposure to Aβ 1–42 fibrils, Aβ 14–23 fibrils, or Aβ 1–42 fibrils formed while co-incubated with Aβ 14–23 (n = 3, * p < 0.05m *** p < 0.001, **** p < 0.0001). (PDF) [file pone.0129087.s001.pdf]

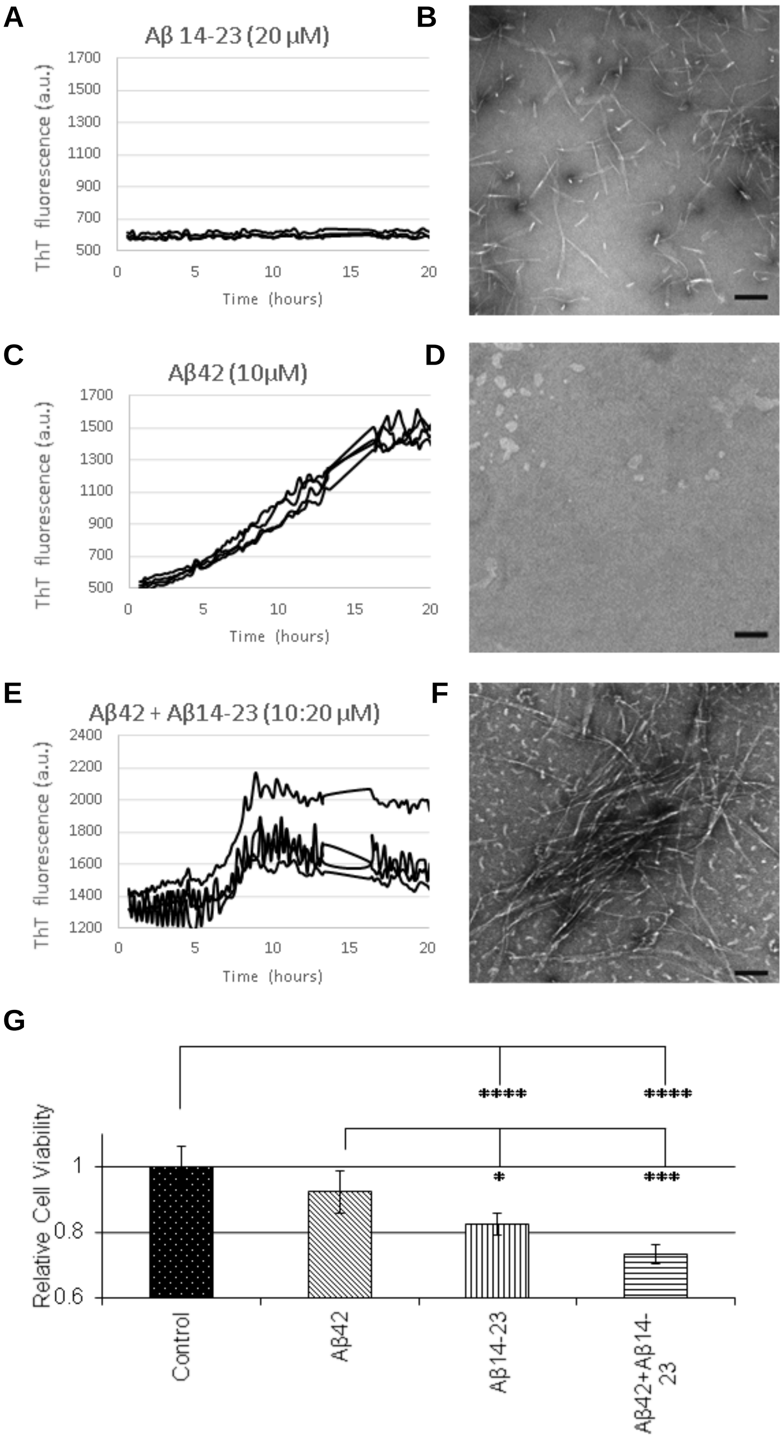

Figure S1. Fibril characteristics and toxicity of Aβ 14-23 peptide (a control peptide composed of all L amino acids). ThT fluorescence kinetics and EM of samples from the starting time of the reaction are shown for Aβ 14-23 (A and B), Aβ 1-42 alone (C and D), and Aβ 1-42 in the presence of Aβ 14-23 (E and F). (G) SHSY-5Y cell viability after 48 hours of exposure to Aβ 1-42 fibrils, Aβ 14-23 fibrils, or Aβ 1-42 fibrils formed while co-incubated with Aβ 14-23 (n=3, * $p < 0.05$m *** $p < 0.001$, **** $p < 0.0001$).
