# Supplementary material for: Structure-Based Peptide Design to Modulate Amyloid Beta Aggregation and Reduce Cytotoxicity
Source: PLoS One. 2015 Jun 12;10(6):e0129087. doi: 10.1371/journal.pone.0129087 (PMC4466325; doi:10.1371/journal.pone.0129087)
Supplement: S2 Fig — (PDF) [file pone.0129087.s002.pdf]

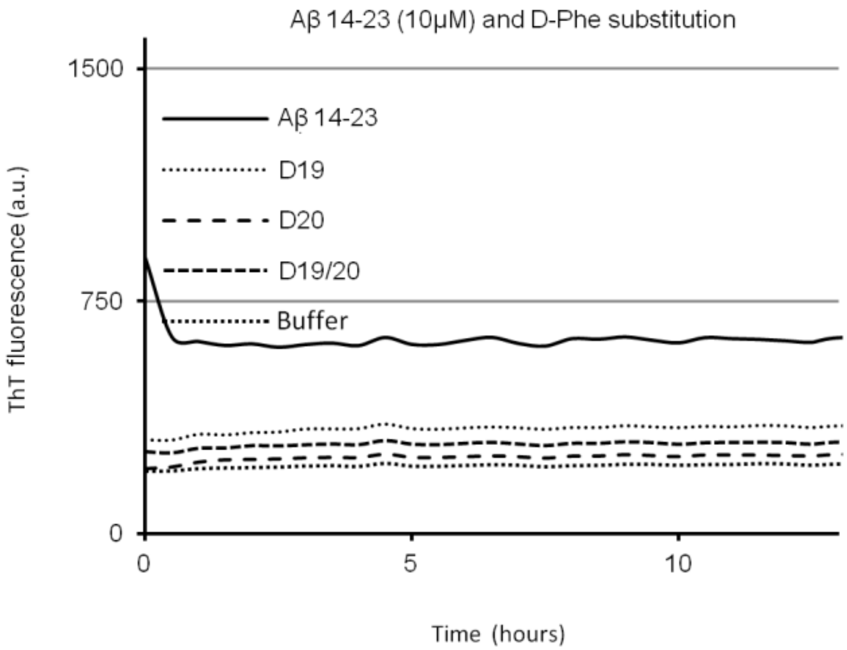

Figure S2. Thioflavin T fluorescence kinetic curves for peptides under fibril forming conditions.
