# Supplementary material for: Structure-Based Peptide Design to Modulate Amyloid Beta Aggregation and Reduce Cytotoxicity
Source: PLoS One. 2015 Jun 12;10(6):e0129087. doi: 10.1371/journal.pone.0129087 (PMC4466325; doi:10.1371/journal.pone.0129087)
Supplement: S3 Fig — (A-C) Reactions were carried out in the presence of 5 μM Aβ 1–42 plus variable ratios of the peptides. (D) A linear increase in lag time is seen with increasing peptide concentration. (PDF) [file pone.0129087.s003.pdf]

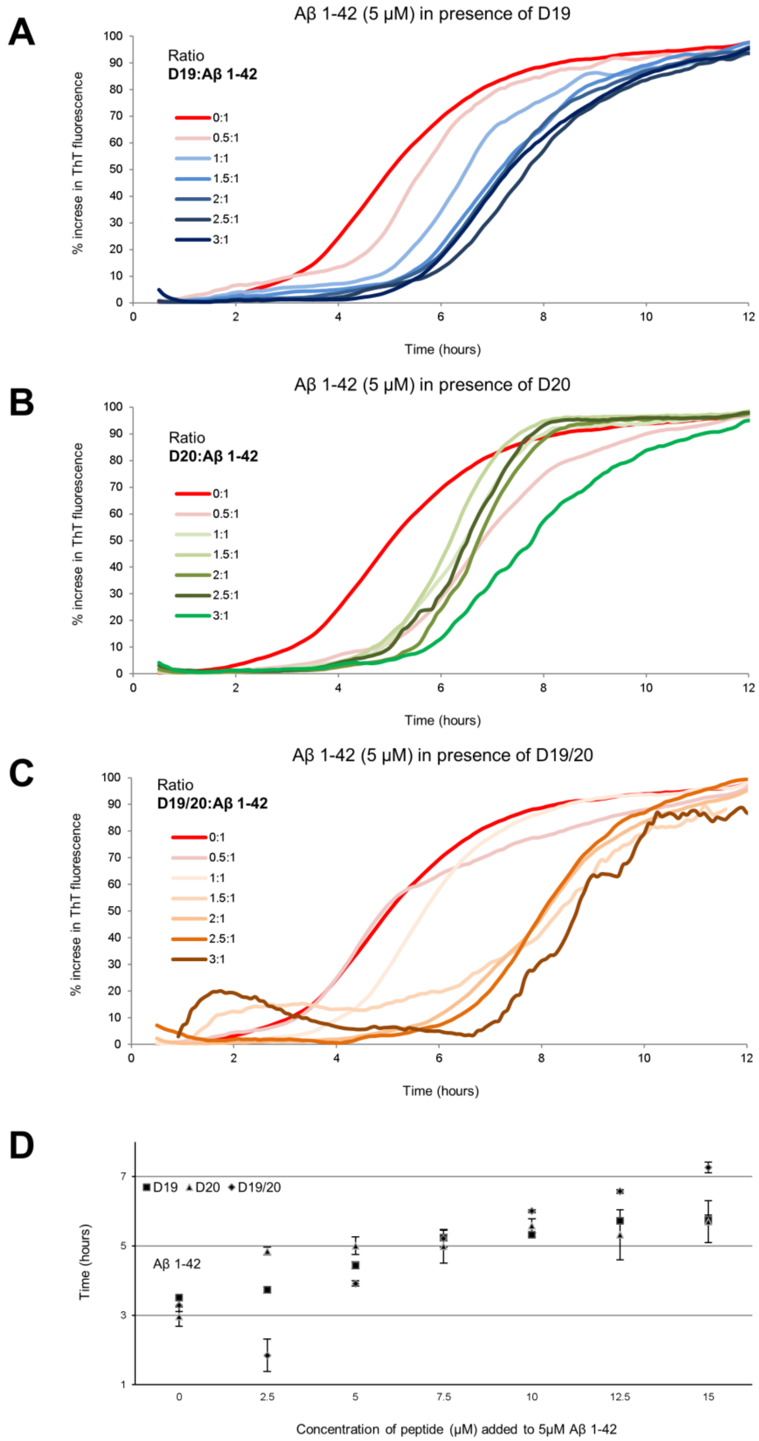

Figure S3. Dose-dependent thioflavin T fluorescence kinetic curves for Aβ 1-42 plus peptides under fibril forming conditions. (A-C) Reactions were carried out in the presence of 5 μM Aβ 1-42 plus variable ratios of the peptides. (D) A linear increase in lag time is seen with increasing peptide concentration.
