# Supplementary material for: Structure-Based Peptide Design to Modulate Amyloid Beta Aggregation and Reduce Cytotoxicity
Source: PLoS One. 2015 Jun 12;10(6):e0129087. doi: 10.1371/journal.pone.0129087 (PMC4466325; doi:10.1371/journal.pone.0129087)
Supplement: S4 Fig — (A) Aβ 1–42 (10 μM) with 1, 2 and 4 fold molar excess of D20 peptide. (B) Aβ 1–42 (2.5 μM) with 8 and 32 fold molar excess of D20 peptide. (PDF) [file pone.0129087.s004.pdf]

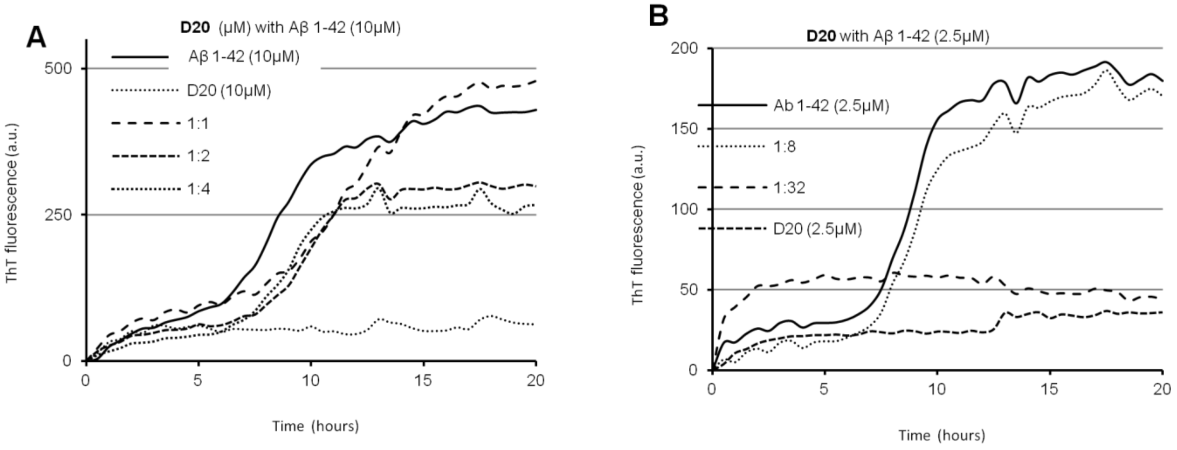

Figure S4. Thioflavin T fluorescence kinetic curves of Aβ 1-42 in the presence of D20 peptide. (A) Aβ 1-42 (10 µM) with 1, 2 and 4 fold molar excess of D20 peptide. (B) Aβ 1-42 (2.5 µM) with 8 and 32 fold molar excess of D20 peptide.
