# Supplementary material for: Structure-Based Peptide Design to Modulate Amyloid Beta Aggregation and Reduce Cytotoxicity
Source: PLoS One. 2015 Jun 12;10(6):e0129087. doi: 10.1371/journal.pone.0129087 (PMC4466325; doi:10.1371/journal.pone.0129087)
Supplement: S5 Fig — Primary cortical neurons were treated with oligomeric preparations of the peptides for 48 hours. The cell viability (by MTS assay) after exposure to Aβ 1–42 oligomers was averaged and normalized to 1 for all experiments. Viabilities for each peptide were normalized to the control Aβ 1–42 values for all experiments and then averaged across experiments. n ≥ 6, *** p < 0.001, **** p < 0.0001. (PDF) [file pone.0129087.s005.pdf]

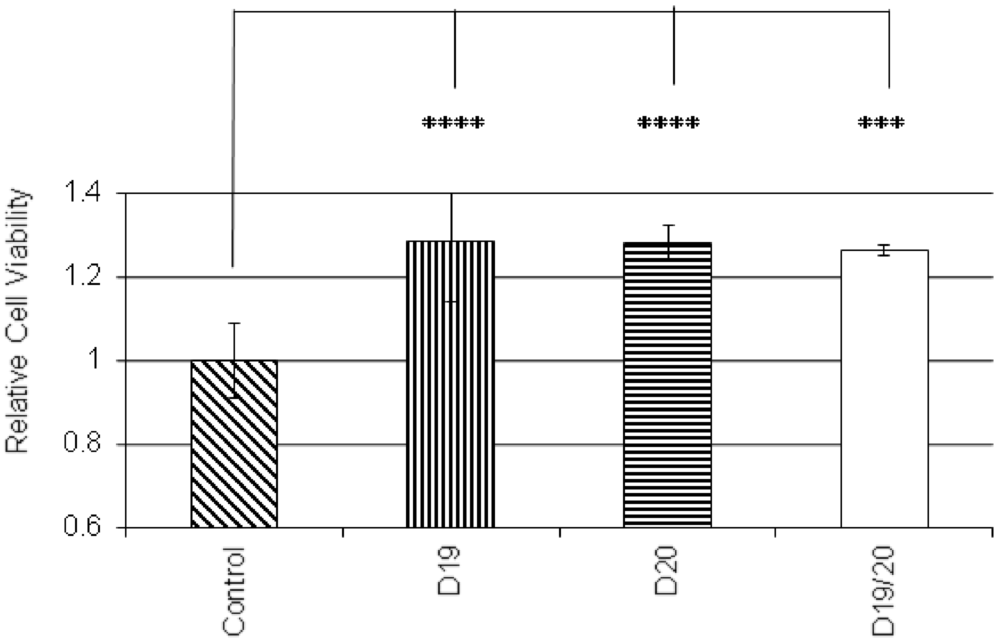

Figure S5. Cell viability after exposure to oligomer preparations of D19, D20 and D19/20, as compared to Aβ 1-42 oligomer exposure. Primary cortical neurons were treated with oligomeric preparations of the peptides for 48 hours. The cell viability (by MTS assay) after exposure to Aβ 1-42 oligomers was averaged and normalized to 1 for all experiments. Viabilities for each peptide were normalized to the control Aβ 1-42 values for all experiments and then averaged across experiments. $n \geq 6$, *** $p < 0.001$, **** $p < 0.0001$.
