# Supplementary material for: Structure-Based Peptide Design to Modulate Amyloid Beta Aggregation and Reduce Cytotoxicity
Source: PLoS One. 2015 Jun 12;10(6):e0129087. doi: 10.1371/journal.pone.0129087 (PMC4466325; doi:10.1371/journal.pone.0129087)
Supplement: S6 Fig — Cells were exposed for 48 hours to Aβ 1–42 fibrils, peptides incubated in fibril forming conditions, or Aβ 1–42 fibrils formed through co-incubation with peptides (Aβ 1–42 + peptide). Significant rescue was observed for all co-incubated samples, compared to Aβ 1–42 fibrils alone. n ≥ 6, *** p < 0.001, **** p < 0.0001. (PDF) [file pone.0129087.s006.pdf]

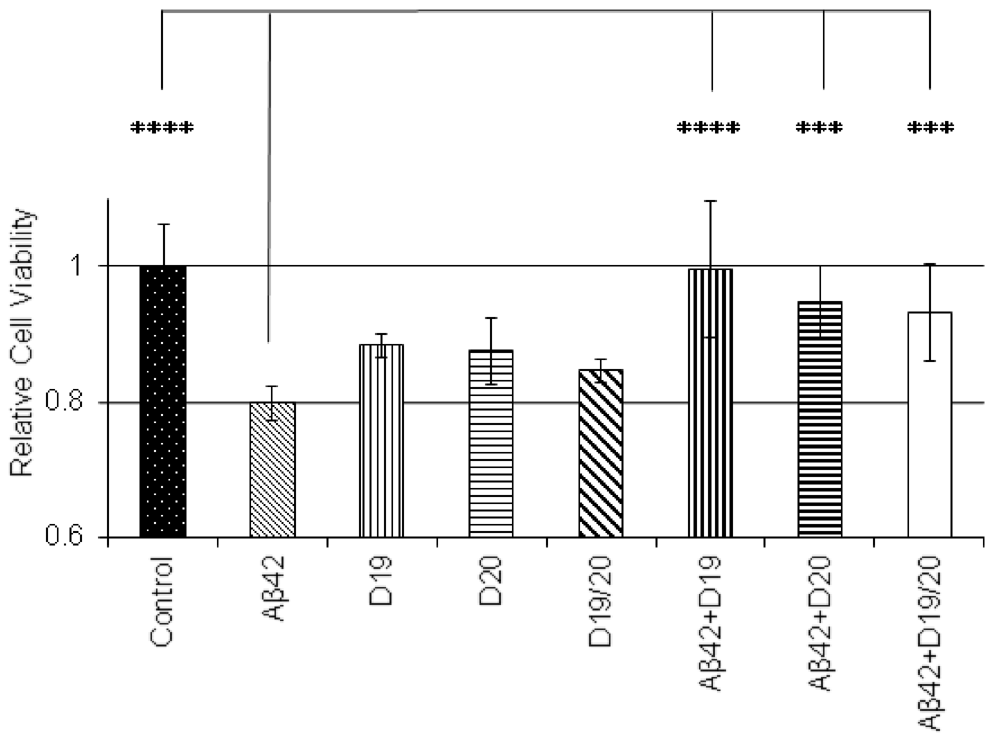

Figure S6. Rescue from Aβ 1-42 fibril-induced cell toxicity as observed in SH-SY5Y cells. Cells were exposed for 48 hours to Aβ 1-42 fibrils, peptides incubated in fibril forming conditions, or Aβ 1-42 fibrils formed through co-incubation with peptides (Aβ 1-42 + peptide). Significant rescue was observed for all co-incubated samples, compared to Aβ 1-42 fibrils alone. $n \geq 6$, *** $p < 0.001$, **** $p < 0.0001$.
