# Supplementary material for: Structure-Based Peptide Design to Modulate Amyloid Beta Aggregation and Reduce Cytotoxicity
Source: PLoS One. 2015 Jun 12;10(6):e0129087. doi: 10.1371/journal.pone.0129087 (PMC4466325; doi:10.1371/journal.pone.0129087)
Supplement: S1 Table — (PDF) [file pone.0129087.s007.pdf]

**Table S7: Summary of relative increases in the lag times seen across all experiments.**

| | Aβ42 | Aβ42+2xD19 | Aβ42+2xD19/20 | Aβ42+2xD20 |
|---|---|---|---|---|
| | 0.95 | 1.38 | 1.11 | 1.16 |
| | 1.05 | 1.59 | 1.08 | 1.38 |
| | 0.62 | 1.56 | 1.82 | 2.19 |
| | 1.06 | 4.38 | 1.81 | 2.23 |
| | 1.32 | 2.91 | 2.00 | 1.81 |
| | 0.94 | 1.16 | 1.11 | 1.11 |
| | 1.04 | 0.91 | 0.95 | 1.78 |
| | 1.02 | 1.23 | 1.13 | 1.62 |
| | 0.81 | 1.23 | 1.74 | 1.62 |
| | 1.16 | 1.12 | 1.44 | 0.87 |
| | 1.03 | 1.13 | 1.15 | 0.88 |
| | 0.34 | 1.42 | 1.35 | 0.85 |
| | 1.33 | 2.21 | 1.15 | 1.46 |
| | 0.85 | 1.96 | 1.09 | 0.90 |
| | 1.48 | 1.25 | 1.10 | 0.98 |
| | 0.95 | 0.74 | 0.78 | 1.21 |
| | 0.94 | 3.66 | 2.90 | |
| | 0.99 | 2.27 | 0.83 | |
| | 1.11 | 1.23 | | |
| | 1.19 | 1.44 | | |
| | 0.81 | | | |
| | 1.02 | | | |
| **Average** | **1.00** | **1.74** | **1.36** | **1.38** |
| **SD** | **0.24** | **0.94** | **0.52** | **0.46** |
| **n** | **22** | **16** | **17** | **15** |
